# A model for design of online health professions education faculty development courses in sub-Saharan Africa

**DOI:** 10.1186/s12909-023-04039-0

**Published:** 2023-01-25

**Authors:** L. Keiller, C. N. Nyoni, C. Van Wyk

**Affiliations:** 1 Division of Health Sciences Education, Office of the Dean, Faculty of Health Sciences, University of the Free State, 205 Nelson Mandela Dr, Park West, Bloemfontein, 9301 South Africa; 2 School of Nursing, University of the Free State, Bloemfontein, 9301 South Africa

**Keywords:** Health professions, Online, Faculty development, Educational design research, Heuristic evaluation, Sub-Saharan Africa

## Abstract

The design of faculty development courses requires a contextually aware theoretical foundation. Therefore, the study aimed to determine the feasibility of a specific conjecture map as a model for designing online health professions education faculty development courses in sub-Saharan Africa through a heuristic evaluation approach. The authors hypothesise that using heuristic evaluation strengthens an educational model's theoretical basis and feasibility.

Twenty-five health professions educators from nine sub-Saharan African countries participated in this multi-phased study. In the first phase, the participants completed a survey evaluating the model and the accompanying author-generated heuristics. Then, participants' recommendations were used to make revisions. In the subsequent phase, participants reviewed these revisions and commented on the feasibility of the model within their local context.

All six heuristics were revised following the initial phase, where 80 problems had been identified. Upon revision, the model was deemed feasible by all except one participant. There was a strong relationship and inter-rater agreement of feasibility between 0.84 and 0.95 regarding the model's practicality, demand, acceptability, and adaptability. Revisions to the final model and guidance documents incorporated all changes the participants recommended, confirming the model's feasibility.

Theoretical models are often developed through a top-down approach, omitting the practice-based considerations that could change the formulation of the model. This study demonstrates the convergence of a theoretical and process model with theoretical, expert and end-user data triangulation. Further research is needed to test this empirically developed model.

**Supplementary Information:**

The online version contains supplementary material available at 10.1186/s12909-023-04039-0.

## Background

Whether in person or online, faculty development courses in health professions education (HPE) aim to facilitate professional growth in teaching, leadership and management skills [1, 2]. The design of effective faculty development courses must adopt evidence-based approaches [3]. Designing online faculty development courses for HPE must consider contextual factors relevant to geographic location [4]. Factors, including funding limitations, pressing health priorities and access, are not uniform across low- and middle-income countries [5]. Regardless, the theoretical and contextual grounding of the design of online courses has not received significant attention [3, 6, 7].

The evidence on the implementation and success of online faculty development courses for HPE in sub-Saharan Africa does not fully incorporate the local geographical gaze [8] and omits the aforementioned contextual factors [6, 7, 9, 10]. This study argues that models for designing faculty development courses should enhance their usability and feasibility [2, 11]. Usability relates to the ability of the proposed system to support the learning process towards desired outcomes [12], while feasibility relates to the practicality of the proposed system within context [13]. The contributions of local experts and end-users increase the courses' contextual relevance, structure, content and outcomes [14, 15].

Models for online learning have been re-developed over decades [16]. All models evolve from hypothetical structures [17] to specific structures within contexts. The feasibility of implementing models requires clarity and alignment to context-specific issues such as funding decisions, curricular decisions and technology [6]. While the authors are aware of the implications for low-and-middle-income countries being strongly linked to developing a community of practice [6], the nuances in these implied geographical contexts are likely to be varied. Therefore, the design of models should embrace empirical evidence of the educational value in context to ensure that both researchers and faculty development practitioners can address the issues mentioned above [18].

Design-based research is one approach that promotes the achievement of theoretical and contextual relevance in educational model design to enhance usability and feasibility [19]. Adopting this scholarly approach [11, 15] supports the feasibility of implementing the designed model. Design-based research allows researchers to conduct empirical research in a naturalistic setting, emphasising local interpretation and context [20]. In a practical sense, both expert and end-user inputs are incorporated in exploring the usability and feasibility of a model. Conjecture mapping is a design-based research method that acts as a conceptualisation mechanism for research, identifying the salient theoretical and design principles applicable to a particular learning environment and mapping the predicted outcomes to these principles [19]. As per design-based research methodology, model development often includes end-user testing for feasibility prior to implementation [21].

In this case, testing for feasibility would require presenting a set of heuristics to educators who design and deliver online HPE faculty development courses [22]. Heuristics are rules of thumb or principles that support the practical enactment of an artefact or a model. The designer generates the heuristics in feasibility testing as a mechanism through which usability problems can be identified by the potential users [23]. The authors developed a set of heuristics and a conjecture map by following a modified Delphi method in a separate study as a precursor to this research (in press). In this study, a specifically designed conjecture map is tested for usability and feasibility as a precursor to implementation.

The feasibility of a designed theoretical model requires the insight and experience of the potential end-users, such as the health professions educators within the geographic region. The study aimed to determine the usability and feasibility of a specific conjecture map as a model for the design of online HPE faculty development courses in sub-Saharan Africa.

## Methods

This study was conducted in multiple phases. This article reports on the final phase of the study. During the pre-study phase, the authors built a conjecture map hypothesising that the salient theoretical and design factors required for online HPE faculty development would be informed by the conversational framework [24] and community of inquiry [25]. Next, three independent studies were conducted to develop the model for designing online courses. In the initial theory-building study, the authors conducted a rapid realist review testing the pre-designed conjecture map to identify the context, mechanism and outcomes of such courses in low and middle-income countries [6]. Using the data from this review, the authors refined the map to reflect six triggering events identified from the results for positive outcomes. These events were programme type, programme design, discussion, engagement, development and collaboration, all reliant on building a community of practice underpinned by the community of inquiry framework [6].

Following the rapid realist review, the revised conjecture map was presented to nine experts in HPE based in sub-Saharan Africa through a modified Delphi study (in press). The panel reached consensus on the identified components of the map and suggested refinement on aspects related to the design and delivery of courses. Findings from this study showed that specific curricular, research, environmental and learning activity types were the components required for the successful design and delivery of online courses. This modified Delphi study informed further adaptation of the map. The final conjecture map was prepared as a process model alongside a set of heuristics that combine theory and practice-based information instead of an exclusively theoretical model [26].

### Design

An adaptation of the process model of the participatory heuristic evaluation approach [23] was applied to explore the usability and feasibility of the model. Traditionally, heuristic evaluations are utilised in software development [22]. Using this approach, the product designers elicit feedback from experts and representative end-users on potential usability problems through independent and collective interactions with the software and designers, respectively [23, 27]. Therefore, the heuristic evaluation was adapted to a model within this study instead of a software product. Additionally, as experts had been included in the preceding modified Delphi study, this heuristic evaluation elicited input from the representatives of the HPE community who are likely to use the model. The participants were thus ideally positioned to provide relevant and contextual input before implementing the promoted model.

### Study population

The authors received gatekeeper permission to contact members of a non-profit organisation that focuses on capacity building in the field of HPE. The members of this organisation are health professions educators in sub-Saharan Africa who have completed a fellowship in HPE and leadership [28]. These members represented the end-users of the proposed model. Two hundred and nineteen members were contacted via email to request participation in the study. Thirty affirmative responses were received.

The authors reviewed the participant list to ensure diversity in regional distribution across sub-Saharan Africa. An additional four participants who were not part of the non-profit organisation, but were experienced and working in the field of HPE, were identified through snowball sampling. These participants represented an underrepresented region in sub-Saharan Africa among the original participant population. Finally, three participants who consented to participate in the study were excluded based on their classification as experts during the preceding phases of this research and thus, not the end-users targeted in this study.

### Data collection

**Fig. 1:**
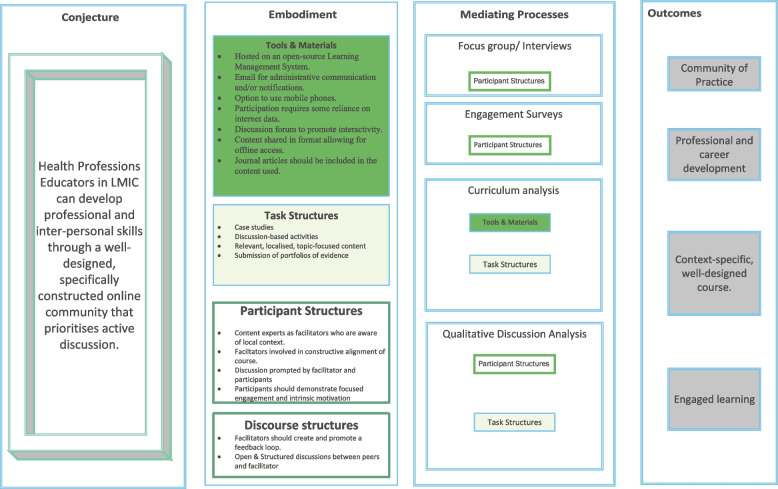
Initial conjecture map

Study data were collected and managed using REDCap electronic data capture tools hosted at the University of the Free State [29, 30]. The participants provided informed consent for participation in this ethics-approved study (UFS-HSD2020/1516/2411). Data were collected in two phases. In phase one, demographic data related to age, gender, number of years as an educator, country of residence and professional position was collected for each participant. In addition, participants were instructed to review the conjecture map as a model (Fig. 1) alongside a set of heuristics with indicators and guiding questions concerning their local context.

The heuristics were developed based on the findings of the modified Delphi study completed in the initial phase, foregrounding the expert opinion from sub-Saharan Africa. The heuristics set for this model were as follows:

1. Systems should be in place to support the sustainability of Online Faculty Development.
2. Tools should be simple, open-access and facilitate discussion.
3. Activities should facilitate engagement and building a Community of Practice.
4. Characteristics of facilitators should be carefully identified to ensure that clinicians and educators are supported.
5. Course design should include planning for evaluation research into the success of the intervention.
6. The Online Faculty Development Programme should have measurable outputs related to professional and personal development..

For each heuristic, participants were asked to confirm the relevance and clarity of the statement provided about the model. Participants were further asked to indicate their agreement with the indicators and guiding questions or make recommendations for changes.

In phase two, participants were asked to comment on the feasibility of the model for designing a contextually relevant online HPE faculty development course in terms of adaptability, demand, acceptability and practicality [31]. The original and changed model was shared online, explaining the changes. The participants were asked to comment on the model's feasibility and revised heuristics.

### Data analysis

Following the first phase of data collection, the first author reviewed the responses and analysed the number of areas of concern identified by the participants. The analysis was done per country, heuristic and geographical region. Next, the authors revised all six heuristics using the recommendations provided by the participants. This revision combined all indicators with the heuristics and refined the guiding questions used for participants in the first phase. The changes were made based on the evaluation, ensuring that non-applicable components were omitted.

In phase two, the revised heuristics and model were presented to the participants in a follow-up survey to determine feasibility within their contexts. A custom-designed scoresheet was used to analyse the level of agreement for each type of feasibility. Agreement was allocated a score of 2, with uncertainty given a score of 1. The highest score possible for each feasibility question per participant was 8. In addition, scores were analysed for inter-rater agreement and feasibility categories compared for correlation to the category with the lowest inter-rater agreement [32]. Finally, the authors analysed the results of the feasibility survey to generate the final model for the design and development of online HPE faculty development courses in sub-Saharan Africa.

## Results

Two hundred twenty-three invitations were sent for study participation, achieving a response rate of 11.2% (n = 25). Distribution by geographic region enabled representation across the participants from all sub-Saharan African regions. Participant representation and demographic data identified nine countries and nine professional disciplines with an average of 16 years of experience as an educator.

Participants identified usability problems in five areas: relevance to their context, clarity, formulation of the heuristic, indicators, and guiding questions. The participants identified 80 problems indicated in red or blue for each record. Green indicates no problems identified. Colour indicators in the total column refer to problems for each heuristic in successive order. (Table 1). The heuristic with the most usability problems was related to the characteristics of the facilitator and participant.

Heuristics and indicators were combined, and guiding questions were revised based on the feedback from participants (Additional file 1).

**Fig. 2:**
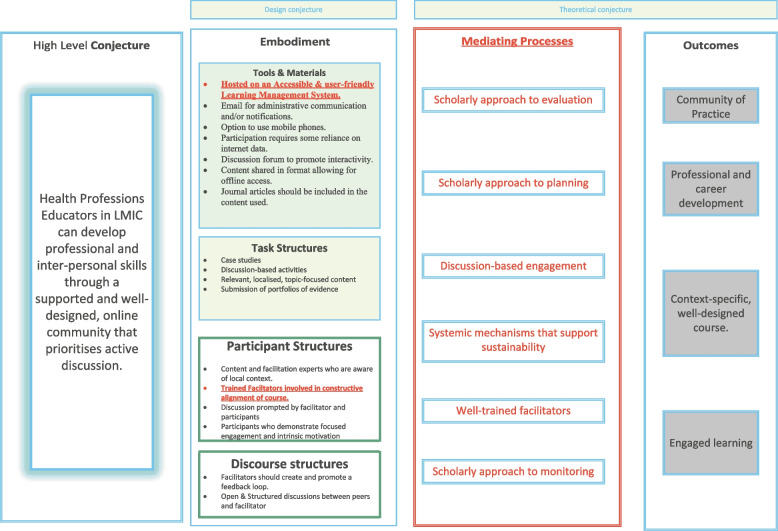
Revision of conjecture map (Revisions in red)

Changes to the model reflected the qualitative recommendations and comments by participants. (Fig. 2).

Feasibility was measured in acceptability, adaptability, demand, and practicality [31] with the feasibility round sent to the twenty-five participants from round 1. A response rate of 76% (n = 19) was achieved. Representation in the responses included eight countries in sub-Saharan Africa and seven professional disciplines. The model was deemed feasible by participants in seven countries and six professional disciplines where feasibility scores demonstrated agreement and a high inter-rater agreement.

The data shows a high level of inter-rater agreement across all participants for all categories, with the lowest being Practicality at 0.84 (Additional File 2).

Demand and Practicality categories demonstrate a strong positive relationship, with Acceptability and Adaptability showing a very strong positive relationship between these categories and demand (Additional File 2) [33].

**Fig. 3:**
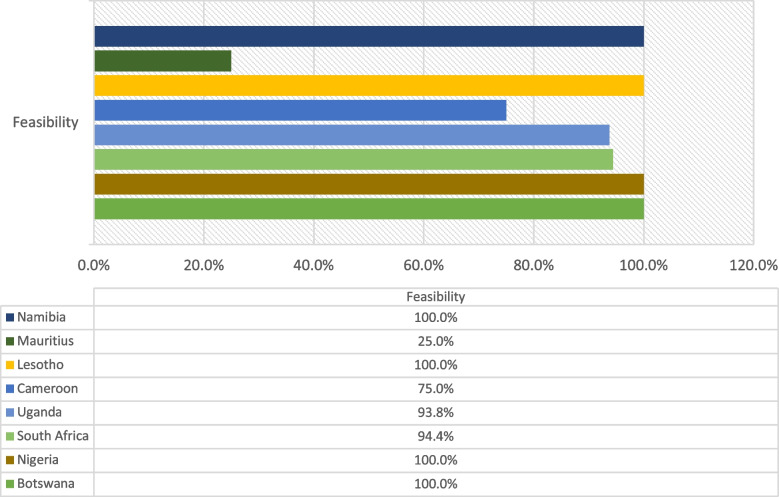
Feasibility per country

**Fig. 4:**
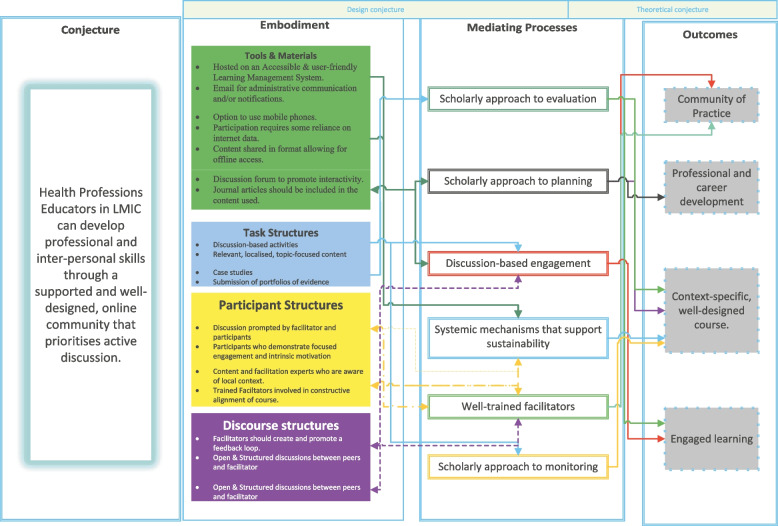
A model for online faculty development courses for health professions education

Upon conclusion of the data analysis, the authors were able to finalise the model with minor adaptations (Fig. 3). In addition, relational arrows were added to indicate the relationship between the design and theoretical conjectures within this model (Fig. 4).

The heuristics deemed usable and feasible by the participants, Systems, Technology, Activities, Characteristics, Evaluation and Outcomes (STACEO) provide the inception point for the administration, design, implementation, and evaluation of online HPE faculty development courses using scholarly approaches (Table 2).

**Table 1:**
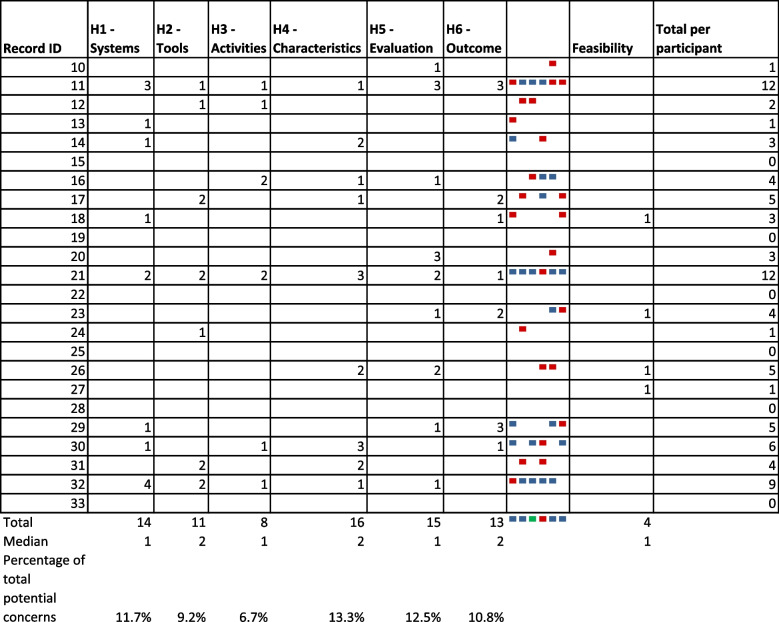
Usability areas of concern

**Table 2:** STACEO Rules for online courses for faculty development in the health professions

Heuristics for online faculty development courses for health professions education	
**S**ystems	Systemic mechanisms exist to support online faculty development's successful planning, implementation, and sustainability
**T**echnology	The technology used facilitates active discussion in a user-friendly and accessible environment
**A**ctivities	Online faculty development incorporates activities that facilitate the formation of a community of educators in which evidence of engagement and development of knowledge, skills and attitudes are prioritised
**C**haracteristics	Well-trained facilitators apply their skills to facilitate constructive dialogue, create a feedback loop, and contribute to the online faculty development course design
**E**valuation	Online faculty development courses follow a qualitative-dominant scholarly approach in design and evaluation to determine short- and long-term outcomes in participants
**O**utcomes	Online faculty development courses have measurable professional and personal development outcomes for facilitators and participants

## Discussion

Theoretical models are often developed through a top-down approach, omitting the practice-based considerations that could change the formulation of the model [26]. This study demonstrates the convergence of a theoretical and process model with the triangulation of theoretical, expert and end-user data in determining the usability and feasibility of a model. In addition, the varied interdisciplinary experience and expertise support the feasibility of the model based on the above evidence. With a combined average of 16 years of experience in HPE, the authors believe that the participants' responses are supported by their experience in faculty development [14]. Furthermore, these participants' varied experience, whether on the ground level or in management positions, provided contextual reference points for them in engaging with the model.

Online faculty development requires evidence to ensure sustainability and efficacy to support the development of skills in research, teaching, leadership and administration [2, 3, 7]. However, the current literature highlights the lack of contextualised approaches to this practice, particularly in low and middle-income countries [6]. The context in developing online learning opportunities does matter [11, 14, 15, 34]. The literature indicates faculty development courses' design as mainly originating from high-income countries [6, 7]. More specifically, participants experience these from the perspective of a foreign or external stakeholder, that is, a foreign gaze [8]. Often, this gaze is further removed from the recipients as the gaze of the funder or provider [35, 36]. By presenting a user-friendly, feasible model for sub-Saharan Africa, the authors contribute to the discussion of the evidence for designing online courses for health professions educators.

Adaptation of standard heuristics for suitability to a specific discipline is encouraged within the field of e-learning [27] and, to our knowledge, has not been adopted within the field of model design in face to face or online faculty development. Therefore, the authors have presented a novel approach that could be included in designing faculty development models, not just as a design-based research methodology [21], but for the scholarly advancement of faculty development. Through the positive findings of this study and the resultant model, the goal of the heuristic evaluation was achieved [37]. That is, a usable and adaptable model within the intended context of HPE in sub-Saharan Africa [22, 23, 37, 38].

The model's policy and structural features had the highest number of identified problems. This outcome resonates with previous research on the feasibility of educational models for online learning [3, 11, 39, 40]. Surprisingly, the identified problems were related to the semantics or combination of the guiding questions, as opposed to the constructs in a traditional pragmatic evaluation [23]. The authors propose that this outcome indicates the rigour with which the model had been developed, thus meeting the need for a usable model with minor adaptations. Furthermore, the absence of substantive changes to the model demonstrates the internal validity of the input during the first phase [26]. While not unprecedented, this alignment between experts and end-users demonstrates a unique occurrence in heuristic evaluation [38].

Regarding the systems and tools, the participants highlighted issues related to systemic or institutional responsibilities for infrastructure and embedded organisational practice in the form of committees and high-level support. This raises the issue of limited local resources available to create these supportive resources and systems in the region [3, 4, 7, 14]. Regarding technology, the model and heuristics presented initially referred to open-source software. However, the participants' contributions echoed that of international research in that open-source software is not a requirement if user-friendly, engaging software options are available within the organisation presenting the course [3, 6, 41, 42]. The assertion of this critical systemic support by participants should be noted by administrators and leaders of institutions in strategic planning and support. Therefore, ensuring that systems and support structures are in place should be read as a non-negotiable within this model.

Online courses should include case studies, discussion-based activities, and the submission of a portfolio of evidence. Though not specific regarding content within the model, these course activities require that facilitators are competent in either online learning or the subject matter. Including this component in the model aligns with the findings from the literature that a combination or variation of facilitator competencies is required in online faculty development courses [3, 6, 35, 43, 44]. As such, activities prepared by these facilitators should remain focused and aligned with the outcomes set during the course development. The focus could be on professional growth and innovation in teaching, management, leadership or research skills [11, 40, 45].

The characteristics of facilitators and course participants yielded the highest number of identified problems. This was dominated by identifying a required semantic change, eliminating the separation of clinical and health professions educators within the heuristic. More importantly, the problems identified across the panel for this construct were related to the need for well-trained facilitators, process experts, health professions educators, and disciplinary experts. While appearing to be problem identification, this finding supports the model as it is developed based on the components within the Community of Inquiry [25]. The teaching presence supported by these trained facilitators ensures targeted and engaged learning [46], mainly when constructive feedback is provided through discursive practice prioritisation [6].

Online HPE faculty development studies have focused on participant experience and organisational expectations for the outcome of courses [3]. However, in this study, participants did not focus on the recipients; instead, the focus was on facilitator roles and supportive structures. Again, this finding speaks to the importance of teaching presence as an influential factor in effectively delivering online courses. Therefore, these courses should focus on the structure and process of an educational experience. It should set the climate through an overlap of the teaching and social presence [46–49] in the facilitators' role of creating a community of practice.

There were no disagreements on the feasibility of the model across seven countries in sub-Saharan Africa and six professional disciplines. While the question may be raised as to whether the lack of country diversity in the presence of regional diversity plays a role, we contend, as do others [50], that individual country differences will be minimal within the sub-Saharan African region. Additionally, the volunteer nature of participants whose experience in an HPE fellowship could influence their responses could contribute to this level of agreement. This should be further investigated by applying the model to a specific course within a particular country. Additional feasibility testing using a granular approach across larger representative samples should investigate the model on specific courses in specific countries in sub-Saharan Africa. Finally, it would be prudent to highlight the local gaze within the recommended research concerning funding, policy, and other institutional structures [3, 40, 51].

While the novel collection of this data contributes to future practice, the authors recognise the limitation of a small number of participants, predominantly situated in anglophone Southern Africa, as a threat to usability across sub-Saharan Africa. Nevertheless, the nature of the findings, limited variance in inter-rater agreement and the preceding phases of this study demonstrate the approach taken using a representative sample.

## Conclusion

The methods used in this multi-phased study demonstrate the rigour with which the authors recommend model development be approached. Within any model development, as with the development of educational interventions, the hypothesis could be wrong [16, 20]. In the initial phases, the authors demonstrated the importance of exploring the literature in designing an online faculty development course and further established the findings' authenticity from the literature through expert review from the local context. Finally, before dissemination, the authors sought to determine its feasibility through representatives of the model recipients in sub-Saharan Africa. The authors have empirically tested and designed this model that may be used as a practical guide for educators who wish to design online faculty development courses. This study, therefore, provides health professions educators and faculty development practitioners from sub-Saharan Africa with a theoretically grounded model for online HPE faculty development courses that are contextually relevant, usable, and feasible.

## Supplementary Information

**Additional file 1. **Originaland revised Heuristics.

**Additional file 2. **Inter-rater Agreement. Relationship betweencategories.

## Data Availability

Relevant data and materials have been provided as Additional File 1 and 2, referenced in the manuscript. The datasets analysed during the current study are available from the corresponding author upon request.
