# Supplementary material for: A model for design of online health professions education faculty development courses in sub-Saharan Africa
Source: BMC Med Educ. 2023 Jan 25;23:60. doi: 10.1186/s12909-023-04039-0 (PMC9878743; doi:10.1186/s12909-023-04039-0)
Supplement: Supplementary file 1 — Additional file 1. Originaland revised Heuristics. [file 12909_2023_4039_MOESM1_ESM.docx]

Additional File 1: Original and revised Heuristics

| Initial Evaluation | | | |  | Revised |
| --- | --- | --- | --- | --- | --- |
| Heuristic | Systems should be in place to support the sustainability of Online Faculty Development | **Indicators** | Organisational, cultural/local, and curricular systems are in place to ensure support and sustainability of Online Faculty development.  Support systems for curricular design, facilitation skills and recipients are available.  Technological systems and network access are available within the environment where the course is being offered. | **Heuristic** | Systemic mechanisms exist to support the successful planning, implementation, and sustainability of online faculty development. |
| Guiding questions | Is my organisation able to provide systemic support through departments and formal structures for initiating Faculty Development?  Is there a need for Faculty Development in a particular area to meet a curricular or local requirement?  Are there senior or influential officials capable of supporting Faculty Development efforts to ensure sustainability?  Are there support structures in place to ensure that facilitators and designers are equipped to design a Faculty Development course successfully?  Are there technology systems and network access issues that need to be addressed to ensure that a fully online Faculty Development course is successful? | | | **Guiding questions** | Can my organisation provide systemic support through departments and formal structures such as high-level committees for initiating faculty development?  Is there a need for faculty development in a particular area to meet a curricular or local requirement?  Are there senior or influential officials and policies capable of supporting faculty development efforts to ensure sustainability?  Have you identified champions–motivated educators who can encourage colleagues–to participate in faculty development opportunities?  Are there support structures in place to ensure that facilitators and curriculum developers are equipped to design a faculty development course successfully?  Are there technology systems and network access issues in the organisation that need to be addressed to ensure that a fully online faculty development course is successful? |
| Heuristic | Tools should be simple, open-access and facilitate discussion. | I**ndicators** | A Learning Management System is used to facilitate learning and teaching, focusing on discussion as an engagement tool.  Email is used for administrative purposes and not as a virtual learning environment. | **Heuristic** | The technology used facilitates active discussion in a user-friendly and accessible environment. |
| Guiding questions | Is there a suitable open-access software package or set of packages utilised as a Learning Management System?  Has email been integrated into the virtual learning environment to ensure smooth administration of course matters? | | | **Guiding questions** | Is there a suitable, accessible, user-friendly software package or set of packages that can be utilised as a Learning Management System?  Have training materials and opportunities been made available to facilitators and participants in using the Learning Management System?  Has email been integrated into the virtual learning environment to ensure smooth administration of course matters? |
| Heuristic | Activities should facilitate engagement and building a Community of Practice. | **Indicators** | Presentation of case studies for discussion and as evidence of learning is included in an Online Faculty Development Course.  The facilitator prioritises constructive dialogue for feedback on and for learning.  Participants submit a portfolio of evidence to represent their learning within the course. | **Heuristic** | Online faculty development incorporates activities that facilitate the formation of a community of educators in which evidence of engagement and development of knowledge, skills and attitudes are prioritised. |
| Guiding questions | Have case studies been identified that meet the focus of the course?  Have instructions been set to ensure that participants can adequately prepare and present educational case studies as a part of the course activities?  Has an opportunity for discussion been included throughout the course?  Have the components of a portfolio of evidence been identified and constructively aligned with the course outcomes?  Has the format of a portfolio been finalised?  Has a rubric for assessment and evaluation of the portfolio been established? | | | **Guiding questions** | Have learning activities been designed to ensure equal participation opportunities for all participants?  Do the activities allow for interdisciplinary participation and or interaction if needed?  Have case studies been identified that meet the focus of the course?  Have instructions been set to ensure that participants can adequately prepare and present educational case studies as a part of the course activities?  Has an opportunity for discussion been included throughout the course?  Have the components of a portfolio of evidence been identified?  Has the format of a portfolio been finalised?  Has a rubric for assessment and evaluation of the portfolio been established?  Have the learning activities and evaluation methods been constructively aligned with the course outcomes?  Have suitable evaluators been trained for the participants’ effective evaluation? |
| Heuristic | Characteristics of facilitators should be carefully identified to ensure that clinicians and educators are supported. | **Indicators** | Facilitators are experts in the topic being covered in the course.  Facilitators have the skills required for online facilitation.  Facilitators provide regular constructive feedback to participants.  Educators and clinicians participating in a course understand their responsibility to be self-directed learners.  Participants assume shared responsibility with facilitators to remain engaged with the course being offered. | **Heuristic** | Well-trained facilitators apply their skills in facilitating constructive dialogue, creating a feedback loop, and contributing to the online faculty development course design. |
| Guiding questions | Who are the potential facilitators of the course?  Have the facilitators been appointed based on expertise within the field related to the focus of the course?  What measures are required to ensure that facilitators are adequately skilled in presenting the topic for the course?  Have facilitators had experience in the delivery of online courses?  Do facilitators clearly understand the importance of discursive practices and feedback for online facilitation?  Has the course been designed to ensure that the facilitators provide regular individual and group feedback?  Has the course been advertised to educators and clinical facilitators with a clear outline of their responsibility to engage fully for the duration of the course?  Do participants understand the requirements from a technological perspective?  Have participants received clear instructions within the course design on engagement levels required? | | | **Guiding questions** | Who are the potential facilitators of the course?  Have the facilitators been appointed based on expertise within the field related to the focus of the course?  What measures are required to ensure that facilitators are adequately skilled in presenting the topic for the course?  Have facilitators had experience in the delivery of online courses?  Do facilitators clearly understand the importance of discursive practices and feedback for online facilitation?  Has the course been designed to ensure that the facilitators provide regular individual and group feedback?  Has the course been advertised to educators with a clear outline of their responsibility to engage fully for the duration of the course?  Do participants understand the requirements from a technological perspective?  Have participants received clear instructions within the course design on engagement levels required? |
| Heuristic | Conduct evaluation research into the success of the course. | **Indicators** | Skilled evaluators should evaluate curriculum documents and portfolios.  Focus group discussions and reflective essays evaluate participant experience in a course.  Qualitative descriptive research should be presented locally and internationally on the success or failure of the course to meet the intended outcomes.  Participants should be interviewed and assessed for skill development through the course provided (e.g., pre/post evaluation) | **Heuristic** | Online faculty development courses follow a qualitative-dominant scholarly approach in design and evaluation to determine participants’ short- and long-term outcomes. |
| Guiding questions | Are there skilled evaluators available within the organisation or local context to assess participant artefacts?  Have research outcomes been set with guiding questions for a focus group or individual interviews?  Are participants willing to participate in the scholarly evaluation of the course?  Are there local and national opportunities for dissemination of course evaluation findings?  Are there skilled qualitative researchers available to conduct interviews and create pre-test evaluations? | | | **Guiding questions** | Are skilled evaluators available within the organisation or local context to assess participant artefacts, i.e., portfolios and assignments?  Have you formulated an ethics proposal for the registration of a monitoring and evaluation study on the implementation and outcome of the course?  Have research outcomes been set with guiding questions for a focus group or individual interviews?  Are participants willing to participate in the scholarly evaluation of the course?  Are there local and national opportunities for dissemination of course evaluation findings?  Are there skilled researchers available to conduct interviews and create pre-test evaluations? |
| Heuristic | The Online Faculty Development Programme should have measurable outputs related to Professional and Personal Development. | **Indicators** | Online Health Professions Education Faculty Development Courses are constructively aligned with professional and personal development outcomes. | **Heuristic** | Online faculty development courses have measurable professional and personal development outcomes for facilitators and participants. |
| Guiding questions | Has your course been constructively aligned to ensure professional and personal development? | | | **Guiding questions** | Has your course been constructively aligned to ensure professional and personal development?  Have participants successfully completed the course?  Have participants received a professional promotion and or accolades based on their development through the course?  Are participants able to benefit from networking and collaborative professional opportunities? |
