# Supplementary material for: A model for design of online health professions education faculty development courses in sub-Saharan Africa
Source: BMC Med Educ. 2023 Jan 25;23:60. doi: 10.1186/s12909-023-04039-0 (PMC9878743; doi:10.1186/s12909-023-04039-0)
Supplement: Supplementary file 2 — Additional file 2. Inter-rater Agreement. Relationship betweencategories. [file 12909_2023_4039_MOESM2_ESM.docx]

**Inter-rater Agreement**

| **Acceptability** | **Demand** | **Practicality** | **Adaptability** |
| --- | --- | --- | --- |
| 0.95 | 0.95 | 0.84 | 0.92 |

**Relationship between categories**

| **Feasibility Categories** | **Acceptability and Practicality** | **Demand and Practicality** | **Adaptability and Practicality** |
| --- | --- | --- | --- |
| **Pearson's r** | 0.70 | 0.41 | 0.77 |
